# A  substructure‐aware graph neural network incorporating relation features for drug–drug interaction prediction

**DOI:** 10.1002/qub2.66

**Published:** 2024-07-06

**Authors:** Liangcheng Dong, Baoming Feng, Zengqian Deng, Jinlong Wang, Peihao Ni, Yuanyuan Zhang

**Affiliations:** ^1^ School of Information and Control Engineering Qingdao University of Technology Qingdao China

**Keywords:** drug–drug interaction, relation features, self‐adaptive pooling

## Abstract

Identifying drug–drug interactions (DDIs) is an important aspect of drug design research, and predicting DDIs serves as a crucial guarantee for avoiding potential adverse effects. Current substructure‐based prediction methods still have some limitations: (i) The process of substructure extraction does not fully exploit the graph structure information of drugs, as it only evaluates the importance of different radius substructures from a single perspective. (ii) The process of constructing drug representations has overlooked the significant impact of relation embedding on optimizing drug representations. In this work, we propose a substructure‐aware graph neural network incorporating relation features (RFSA‐DDI) for DDI prediction, which introduces a directed message passing neural network with substructure attention mechanism based on graph self‐adaptive pooling (GSP‐DMPNN) and a substructure‐aware interaction module incorporating relation features (RSAM). GSP‐DMPNN utilizes graph self‐adaptive pooling to comprehensively consider node features and local drug information for adaptive extraction of substructures. RSAM interacts drug features with relation representations to enhance their respective features individually, highlighting substructures that significantly impact predictions. RFSA‐DDI is evaluated on two real‐world datasets. Compared to existing methods, RFSA‐DDI demonstrates certain advantages in both transductive and inductive settings, effectively handling the task of predicting DDIs for unseen drugs and exhibiting good generalization capability. The experimental results show that RFSA‐DDI can effectively capture valuable structural information of drugs more accurately for DDI prediction, and provide more reliable assistance for potential DDIs detection in drug development and treatment stages.

## 1 INTRODUCTION

Polypharmacy is gradually becoming a common pharmacological treatment approach for patients in maintaining their overall physical well‐being, accompanied by challenges in drug safety. When multiple drugs are used concomitantly, interactions between drugs may occur, which may lead to an increased occurrence of adverse reactions [1]. The unexpected interactions that occur during polypharmacy are known as drug–drug interactions (DDIs). As a typical concern during polypharmacy, DDIs are related with roughly 30% of known adverse drug events [2, 3]. Therefore, the identification and assessment of DDIs during drug development and pharmacovigilance have become particularly crucial.

Traditionally, the assessment of DDIs has been accomplished by comprehensive pharmacological analysis and clinical trials. Pharmacological analysis requires in‐depth investigation of the characteristics and mechanisms of action of each drug, involving extensive data analysis. Clinical trials require long‐term monitoring and data collection, and their scale and complexity increase the time and cost of the entire process. Therefore, this approach is intricate and laborious. However, computer‐based methods have the advantage of automating the processing of massive data, utilizing the high‐speed computing and simulation analysis of computers to more accurately predict potential DDIs from known DDIs, accelerating research progress and reducing costs, making it an economically viable alternative. Computer‐based solutions can generally be divided into text mining‐based methods [4, 5, 6, 7] and deep learning (DL)‐based methods [8, 9]. Text mining‐based methods utilize various data sources such as biomedical literature [4, 5, 6], clinical trial data, and the Food and Drug Administration Adverse Event Reporting System [7] to extract implicit drug–drug relations among different entities. However, these methods suffer from problems related to the lack of effective data quality assurance and potential ambiguity in the extracted relations, resulting in a suboptimal prediction effect.

Recently, DL models and graph neural networks (GNNs) have achieved promising results in biomedical research, demonstrating their remarkable potential for applications, particularly in predicting DDIs. The corresponding methods for predicting DDIs can be roughly categorized into network‐based methods and molecular structure‐based methods.

Network‐based methods predominantly leverage the topology of relevant networks and incorporate biomedical data from multiple information sources. They tend to be broadly grouped into graph embedding [10], link prediction [11, 12], and knowledge graph‐based [13, 14] methods. They structure the relevant information into an embedding space, a DDIs network, or a knowledge graph. Then, they integrate multimodal data and employ various graph‐specific techniques such as sparse regularization methods [15] and matrix factorization [16] for searching or inferring to facilitate the prediction of DDIs. However, these methods often heavily rely on the richness and reliability of the data within the network, requiring high levels of integrity, logical consistency, and scale of the information network.

In contrast, molecular structure‐based methods exclusively leverage the chemical structures and characteristics of drugs for prediction without the dependence on external biomedical knowledge. Among them, many studies are based on the assumption of drug similarity [17, 18, 19, 20, 21, 22], which suggests that drugs with similar structural features exhibit similar pharmacological and interaction properties. Multimodal deep learning framework for predicting drug–drug interaction events (DDIMDL) [23] and deep‐learning strategy for interpretable prediction of DDIs that leverages drug‐induced gene expression signatures (DeSIDE‐DDI) [24] respectively design a novel DL framework based on the similarity hypothesis, dynamically aggregating multiple drug features for DDIs prediction. However, the computation complexity for calculating the similarity matrix between drugs is high, especially when dealing with large‐scale datasets. In addition, some works focus on considering the overall chemical structure of drugs [25, 26, 27]. A general graph‐based deep learning framework for drug–drug interactions and drug–target interactions prediction (DeepDrug) [28] utilizes edge feature fusion‐based RGCNs to extract graph structural information of compounds. Multi‐head co‐attentive drug–drug interactions prediction (MHCADDI) [29] employs a co‐attention mechanism to learn latent drug representations in the process of drug overall feature extraction. However, DDIs are fundamentally caused by the interactions of chemical substructures. Researchers have started exploring using GNNs for substructure feature extraction [30, 31, 32] and extending these features to DDIs prediction tasks for other drug pairs. Substructure–substructure interactions for drug–drug interaction prediction (SSI‐DDI) [33] directly decompose the interactions between drugs into pairwise interactions between corresponding substructures to predict potential DDIs. Gated message passing neural network (GMPNN) which learns chemical substructures for DDI prediction (GMPNN‐CS) [34] introduces a GMPNN, which utilizes “gates” (edge weights) to control the extraction of substructures. However, these gates do not fully utilize the structural information of drug molecules. Other works [35, 36, 37, 38] also utilize GNNs for substructure feature learning but similarly only consider the local structural information of drugs without fully exploiting the distinctive details of the nodes.

In general, the present DL methods that extract knowledge from known DDIs to predict potential DDIs have made significant progress. However, there are still some issues that need to be addressed. Firstly, in the process of feature extraction, the commonly used readout functions (e.g., global mean/max pooling) in GNNs fail to effectively utilize both the feature information of nodes and the local information of the graph. As a result, it becomes challenging to extract high‐quality feature representations, which in turn affects the adaptive extraction of substructures and the evaluation of the degree of interaction impact. Furthermore, the above conventional methods have overlooked the impact of specific relation types information on the construction of drug features. They merely randomly initialize the relation embedding and feed it into the prediction module for DDIs projection, which might potentially impair the model’s discriminative performance to some amount.

**FIGURE 1:**
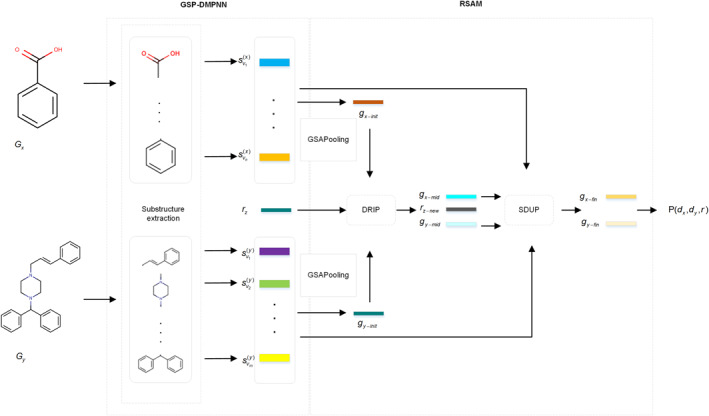
RFSA‐DDI framework. The predicted drug pair and its potential relation type are utilized as the input. Substructure extraction is performed on drug molecule graph by GSP‐DMPNN module. In RSAM module, the substructures are pooled firstly using GSAPooling to get initial graph representation. Then, the DRIP is used to update the graph representation and the relation representation jointly. The SDUP is used to calculate the substructure importance and obtain the final drug representation by weighted aggregation. The final DDI prediction is the interaction probability between the updated relation feature and the final drug representations. DRIP, drug information and relation information interactive perception process; GSAPooling, graph self‐adaptive pooling; GSP‐DMPNN, directed message passing neural network with substructure attention mechanism based on graph self‐adaptive pooling; RSAM, substructure‐aware interaction module incorporating relation features; SDUP, graph‐level feature update process based on substructure‐drug perception.

**FIGURE 2:**
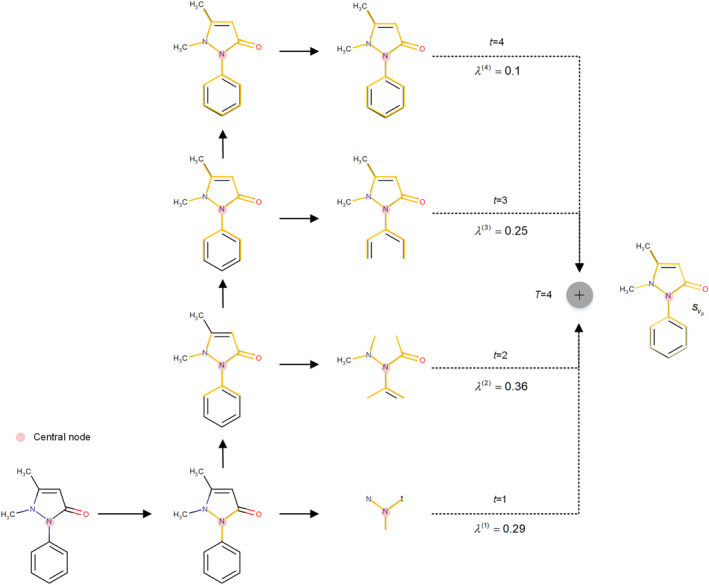
Description of elastic substructure extraction. The original drug (antipyrine) is depicted via a chemical structure diagram. A node (such as the pink central node) is evolved by repeatedly aggregating message from its nearby nodes *T* times with weights *λ* ^(*t*)^. Then, substructures of different sizes and shapes are extracted.

To address the foregoing shortcomings, we offer a model framework named RFSA‐DDI, as shown in Figure 1. Firstly, to extract the hidden features of substructures, we propose a directed MPNN with substructure attention mechanism based on graph self‐adaptive pooling (GSP‐DMPNN). By employing the GSApooling layer [39] for pooling operations, we can integrate the node feature information and local information of drugs, aggregating neighbor key‐level features and generating bond‐level graph representation at each layer. These representations are utilized to compute the importance of atomic substructures with different radii. Thus, a more reasonable molecular substructure feature centered around an atom can be extracted by calculating the weighted sum of different radius substructures, as shown in Figure 2. In addition, we propose a substructure‐aware interaction module incorporating relation features (RSAM), which mainly includes the drug information and relation information interactive perception process (DRIP) and a graph‐level feature update process based on substructure‐drug perception (SDUP). DRIP takes into account the significance of specific relations by incorporating relation features into drug representations and refines them jointly within the process, aiming to improve the relation representations and current representations of drug features. Next, SDUP evaluates the effect of substructures of one drug on the interaction with another drug to identify important substructures involved in DDIs effectively. Finally, we execute weighted aggregation of the drug substructures to generate the final representation of drug. We undertake experiments on both DrugBank and Twosides datasets and discover that RFSA‐DDI demonstrates advantages in predicting DDIs under the transductive setting, showing better robustness and interpretability. When considering only the DrugBank dataset and redefining the negative sample selection range for DDI tuples in the inductive setting, we observe that this approach also achieves good performance.

## 2 RESULTS

### 2.1 Experiment setting

Our model, named RFSA‐DDI, was implemented using PyTorch and PyTorch Geometric. All experiments were conducted on a Windows 10 operating system with an NVIDIA GeForce GTX1080 Ti, an Intel(R) Xeon(R) W‐2133 central processing unit @ 3.60 GHz (12 CPUs), and 32 Gigabyte of RAM. Hyper‐parameters were modified using random search, and the optimal values were determined pursuant to the overall outcomes of the validation set. We investigated the subsequent hyper‐parameter configurations: the total number of epochs was set to 100. The quantity of message passing iterations *T* was modified among {3,5,8,10,15}; the dimension of hidden substructure features $s_{v_{p}}$ was tuned from {64,128}. The *LR* was modified among {1e−2,1e−3,1e−4}. Moreover, an exponentially decaying scheduler of 0.96^*η*^(where *η* represents the latest epoch) was applied to *LR*. We utilized the Adam [40] optimizer to obtain the optimal Batch size of DDI tuples, which was modified among {128,256,512}. After parameter analysis, we found that the combination of *T* = 8, $s_{v_{p}}\in R^{128}$, *LR* = 1e−3, and a Batch size of 256 achieved the best performance. Similar to the majority of the research [37, 41, 42], we tested the comparison methods in the same experimental environments as RFSA‐DDI. The performance metrics included accuracy (ACC), area under the curve (AUC), F1‐score (F1), precision (Prec), recall (Rec) and average precision (AP).

### 2.2 Baselines

We compare our method RFSA‐DDI against the latest baselines which only take the structural information of drugs as input and consider the impact of drug substructures on DDI prediction during the learning process.

1. SSI‐DDI [33]: decomposes the complex task of predicting DDIs into simpler substructure interaction prediction tasks, thereby improving prediction performance.
2. GMPNN‐CS [34]: introduces a GMPNN to capture adaptive‐sized and shaped substructures, where the features of substructures are determined by the updated features of nodes.
3. Substructure‐aware graph neural network for DDI prediction [35]: a directed MPNN with a substructure attention mechanism for capturing substructures of diverse sizes and shapes.
4. Dual graph neural network for DDIs prediction based on molecular structure and interactions [37]: employs dual GNNs to extract features of drugs, focusing on the influential substructures for predicting DDIs. The extraction process comprehensively considers the interaction information between drug substructures and chemical substructures. The method was tested only in the transductive setting using the DrugBank dataset.

### 2.3 Parameter analysis

**FIGURE 3:**
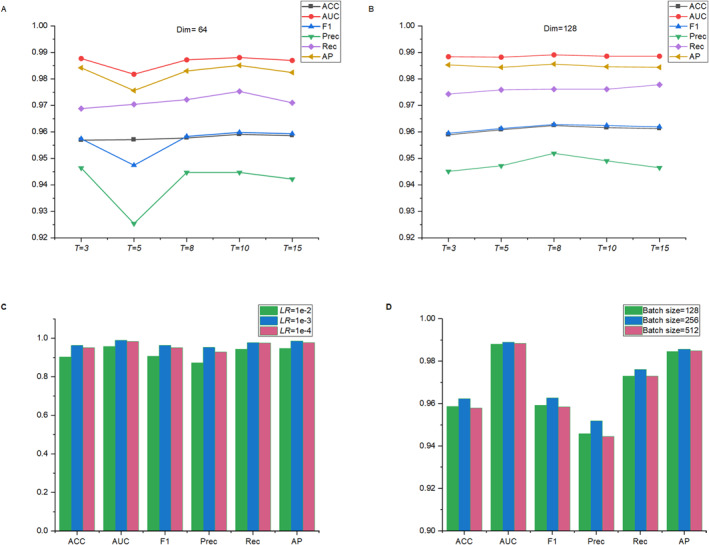
Parameter analysis. (A) and (B) exhibit the impact of the number of iterations *T* and the size of the substructure hidden dimension on the model performance. (C) Demonstrates a direct impact on learning rate. (D) Demonstrates the influence of Batch size. ACC, accuracy; AP, average precision; AUC, area under the curve; Dim, substructure hidden dimension; F1, F1‐score; LR, learning rate; Prec, precision; Rec, recall.

During the feature extraction process, the number of message passing iterations *T* and the dimension of hidden substructure features $s_{v_{p}}$ hold a substantial impact on the level of substructure extraction. We tested all 10 combinations of *T* and $s_{v_{p}}$ on the DrugBank dataset, as shown in Figure 3. The scores for various metrics under all 10 combinations are shown in Figure 3A,B. It can be observed that the model achieves optimal performance when *T* = 8 and the dimension of hidden substructure features $s_{v_{p}}\in R^{128}$. We also investigated the impact of LR and Batch size on model performance, as shown in Figure 3C,D. The LR controls the step size of weight updates during iterations, thereby affecting the convergence speed and precision of the model. The Batch size drastically impacts the training pace and generalization capacity of the model. It can be visually observed that when the *LR* = 1e−3 and the Batch size is 256, the model performance exhibits better performance.

### 2.4 Performance evaluation under the transductive setting

With the transductive setting, the drugs in the test set may have already appeared in the training process, which can test the model’s generalization and memorization capabilities for known drugs. On the DrugBank and Twosides datasets, we partitioned the DDI tuples involved based on interaction type and ensured that the proportions of each interaction type were the same in the training (60%), validation (20%), and test set (20%). We performed this stratified random data subset generation three times and generated an accompanying negative example for each tuple adopting the method given by Wang et al. [43]. To maintain consistency in the training environment, all specimens were constructed before training to guarantee that all methods, including RFSA‐DDI and comparison methods, were trained on the same data and avoided bias issues caused by different samples. The average results obtained by each method in these three experiments are reported in Tables 1 and 2. The optimal result in each metric is indicated in bold.

**TABLE 1:** Comparison results of RFSA‐DDI and baselines on the DrugBank dataset under the transductive setting.

Method	ACC	AUC	F1	Prec	Rec	AP
SSI‐DDI	0.9250	0.9700	0.9285	0.9065	0.9490	0.9620
GMPNN‐CS	0.9430	0.9744	0.9436	0.9256	0.9629	0.9690
SA‐DDI	0.9496	0.9730	0.9501	0.9377	0.9627	0.9711
DGNN‐DDI	0.9510	0.9790	0.9516	0.9376	**0.9755**	**0.9860**
**RFSA‐DDI**	**0.9624**	**0.9891**	**0.9628**	**0.9519**	0.9740	0.9856

*Note*: The optimal result in each metric is indicated in bold.

Abbreviations: ACC, accuracy; AP, average precision; AUC, area under the curve; DGNN‐DDI, dual graph neural network for drug–drug interactions prediction based on molecular structure and interactions; F1, F1‐score; GMPNN‐CS, gated message passing neural network which learns chemical substructures for DDI prediction; Prec, precision; Rec, recall; RFSA‐DDI, substructure‐aware graph neural network incorporating relation features for drug–drug interaction prediction; SA‐DDI, substructure‐aware graph neural network for DDI prediction; SSI‐DDI, substructure–substructure interactions for drug–drug interaction prediction.

**TABLE 2:** Comparison results of RFSA‐DDI and baselines on the Twosides dataset under the transductive setting.

Method	ACC	AUC	F1	Prec	Rec	AP
SSI‐DDI	0.8230	0.8935	0.8301	0.7900	0.8746	0.8615
GMPNN‐CS	0.8580	0.9190	0.8676	0.8115	0.9303	0.8897
SA‐DDI	0.8602	0.9187	0.8681	0.8141	0.9378	0.8950
**RFSA‐DDI**	**0.8715**	**0.9296**	**0.8785**	**0.8265**	**0.9480**	**0.9088**

*Note*: The optimal result in each metric is indicated in bold.

Abbreviations: ACC, accuracy; AP, average precision; AUC, area under the curve; DGNN‐DDI, dual graph neural network for drug–drug interactions prediction based on molecular structure and interactions; F1, F1‐score; GMPNN‐CS, gated message passing neural network which learns chemical substructures for DDI prediction; Prec, precision; Rec, recall; RFSA‐DDI, substructure‐aware graph neural network incorporating relation features for drug–drug interaction prediction; SA‐DDI, substructure‐aware graph neural network for DDI prediction; SSI‐DDI, substructure–substructure interactions for drug–drug interaction prediction.

On the DrugBank dataset, our method delivers the best result in practically all metrics, with only slightly lower scores in terms of Rec and AP. On the Twosides dataset, our method demonstrates excellent performance, getting the optimal in all metrics. Compared to existing methods, our approach exhibits further improvements in performance. These results indicate that our method possesses strong generalization and learning capabilities for known drug structural information, effectively extracting substructure information and accelerating the adaptation and accurate representation of new DDI tuples’ drug features. This enhances DDI prediction performance and can provide better assistance in practical applications such as drug design and safety monitoring.

### 2.5 Performance evaluation under the inductive setting

Different from transductive setting, here, the dataset is separated contingent on drugs, creating a scenario that closely resembles the real‐world use of the model. The inductive setting is more complicated than the transductive setting. In the transductive setting, the model solely works with unseen DDI tuples, but all drugs within the tuples are known during training. In contrast, in the inductive setting, the model needs to handle drugs that were unseen during training, and these drugs may have unknown chemical structures. This adds an additional level of complexity to the task. In this scenario, we arbitrarily choose one‐fifth of the drugs as novel drugs ($D_{\text{new}}$) which are excluded from the training process. DDI tuples comprising two drugs in $D_{\text{new}}$ are extracted into $M_{s1}$, while those comprising two drugs not in $D_{\text{new}}$ are extracted into $M_{\text{train}}$. DDI tuples involving one drug in $D_{\text{new}}$ and one drug not in $D_{\text{new}}$ are collected into $M_{s2}$. This process is reiterated three times to obtain three random subsets of data. Moreover, we have also imposed constraints on the selection range of negative samples. Firstly, for the negative examples in $M_{\text{train}}$, they do not contain drugs that occur in $D_{\text{new}}$. For the negative samples in $M_{s1}$, they only consist of drugs that are present in both $D_{\text{new}}$ and $M_{s1}$. The generation strategy for negative samples in $M_{s2}$ is the same as that of $M_{s1}$. In the inductive setting, the experiments are run utilizing solely the DrugBank dataset because of its comparatively large number of drugs. To prevent the model from overfitting to known drug information during training, we apply regularization techniques and incorporate an additional dropout layer [44].

The average results of the three random fold experiments for all methods are presented in Tables 3 and 4. It can be observed that all models exhibit significant performance degradation compared to the warm‐start scenario. This might be attributed to the substantial structural diversity among drugs in the DrugBank dataset, making it tough for the models to reliably predict DDIs in the lack of sufficient drug structure information. Moreover, the predictive performance on the $M_{s2}$ is better than that on the $M_{s1}$. This is consistent with the fact that the DDI tuples in $M_{s2}$ may contain specific drugs that occur in the training set, leading in the diffusion of associated drug topology details into the test set. However, in comparison, our method exhibits the best overall performance. The predictive performance on the $M_{s1}$ dataset is the best among all methods, and on the $M_{s2}$ dataset, our method achieves high scores in the ACC, AUC, F1, Rec, and AP metrics. This confirms the significant advantage of RFSA‐DDI in better generalizing learned drug substructure information to different drugs with similar substructures, enabling more effective and informative feature representations for drugs and allowing for more accurate prediction of interactions between new and established drugs, as well as between newly developed drugs.

**TABLE 3:** Comparison results of RFSA‐DDI and baselines on the DrugBank dataset under the inductive setting for $M_{s1}$ Partition (new drug—new drug).

Method	ACC	AUC	F1	Prec	Rec	AP
SSI‐DDI	0.5832	0.6534	0.4041	0.7712	0.3423	0.6853
GMPNN‐CS	0.6075	0.6867	0.4266	0.7911	0.2921	0.7067
SA‐DDI	0.6555	0.7355	0.5364	0.8075	0.3987	0.7597
**RFSA‐DDI**	**0.6673**	**0.7600**	**0.5691**	**0.8195**	**0.4394**	**0.7703**

*Note*: The optimal result in each metric is indicated in bold.

Abbreviations: ACC, accuracy; AP, average precision; AUC, area under the curve; DGNN‐DDI, dual graph neural network for drug–drug interactions prediction based on molecular structure and interactions; F1, F1‐score; GMPNN‐CS, gated message passing neural network which learns chemical substructures for DDI prediction; Prec, precision; Rec, recall; RFSA‐DDI, substructure‐aware graph neural network incorporating relation features for drug–drug interaction prediction; SA‐DDI, substructure‐aware graph neural network for DDI prediction; SSI‐DDI, substructure‐substructure interactions for drug–drug interaction prediction.

**TABLE 4:** Comparison results of RFSA‐DDI and baselines on the DrugBank dataset under the inductive setting for $M_{s2}$ Partition (new drug—old drug).

Method	ACC	AUC	F1	Prec	Rec	AP
SSI‐DDI	0.6786	0.7512	0.6378	0.7397	0.5586	0.7563
GMPNN‐CS	0.7218	0.8030	0.6733	0.8153	0.5734	0.8049
SA‐DDI	0.7504	0.8298	0.7093	**0.8492**	0.6090	0.8283
**RFSA‐DDI**	**0.7559**	**0.8327**	**0.7305**	0.8152	**0.6618**	**0.8339**

*Note*: The optimal result in each metric is indicated in bold.

Abbreviations: ACC, accuracy; AP, average precision; AUC, area under the curve; DGNN‐DDI, dual graph neural network for drug–drug interactions prediction based on molecular structure and interactions; F1, F1‐score; GMPNN‐CS, gated message passing neural network which learns chemical substructures for DDI prediction; Prec, precision; Rec, recall; RFSA‐DDI, substructure‐aware graph neural network incorporating relation features for drug–drug interaction prediction; SA‐DDI, substructure‐aware graph neural network for DDI prediction; SSI‐DDI, substructure‐substructure interactions for drug–drug interaction prediction.

### 2.6 Ablation experiments

The improvement in model performance is attributed to the GSP‐DMPNN module and RSAM module, which are dedicated to optimizing the extraction of critical drug substructures and incorporating a relation‐aware process to enhance drug features, intending to get richer and more useful information in the final drug feature representation. To investigate the impact of the GSP‐DMPNN and RSAM modules on model performance, we additionally considered the following variations of RFSA‐DDI:

1. RFSA‐DDI_M: swaps the GSAPooling operation in the GSP‐DMPNN module with global average pooling.
2. RFSA‐DDI_noRA: removes the DRIP process in the RSAM module, which means there is no interactive perception and updating with interaction feature information during the drug feature generation process.
3. RFSA‐DDI_M_noRA: replaces the GSAPooling operation in the GSP‐DMPNN module with global average pooling and removes the DRIP process in the RSAM module.

**FIGURE 4:**
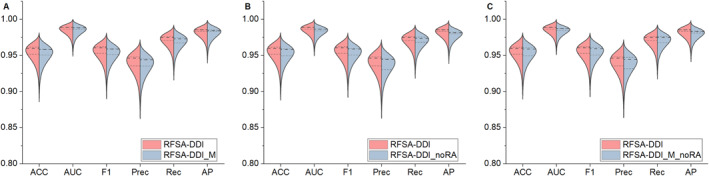
Analysis of the effect of GSP‐DMPNN and RSAM. (A)–(C) show the metric assessments of RFSA‐DDI excluding GSP‐DMPNN and/or RSAM. ACC, accuracy; AP, average precision; AUC, area under the curve; F1, F1‐score; Prec, precision; Rec, recall; RFSA‐DDI, substructure‐aware graph neural network incorporating relation features for drug–drug interaction prediction; RFSA‐DDI_M, RFSA‐DDI replaces graph self‐adaptive pooling with global mean pooling; RFSA‐DDI_M_noRA, RFSA‐DDI replaces graph self‐adaptive pooling with global mean pooling and removes the drug information and relation information interactive perception process in the RSAM module; RFSA‐DDI_noRA, RFSA‐DDI removes the drug information and relation information interactive perception process in the RSAM module.

We conducted experiments on the Drugbank dataset using the same settings as the parameters in our model. Tables 5, 6, 7 summarize the experimental results for transductive setting and inductive setting, respectively. As shown in Table 5, only the Rec metric is slightly lower than other methods in the transductive setting, with a difference of only 0.35% from the highest score in this metric. In the inductive setting, as shown in Tables 6 and 7, most metrics generally exhibit significant improvements compared to other methods. Therefore, we can conclude that the model using GSAPooling pooling outperform the model using average pooling. The performance of the model enhancing drug representation with interaction features surpasses that of the model without it, indicating the significant impact of interaction embeddings on identifying latent drug representation. This also corroborates that both the GSP‐DMPNN module and RSAM module are essential components of the model. To further compare the proposed method with its variants, we used Figure 4 to portray all metric results of these methods in the transductive setting. These violin plots emphasize that RFSA‐DDI exceeds its variants in these metrics. The above data analysis confirms that the combination of the GSAPooling operation and the relation‐aware process does improve DDI prediction performance. The GSAPooling pooling better utilizes drug structure information to identify critical substructures involved in interactions, while relation information optimizes drug features, thus resulting in more informative drug feature representations and improving DDIs prediction effectiveness.

**TABLE 5:** Ablation comparison results of RFSA‐DDI and its deformation on the DrugBank dataset under the transductive setting.

Method	ACC	AUC	F1	Prec	Rec	AP
RFSA‐DDI_M	0.9591	0.9880	0.9597	0.9458	0.9739	0.9842
RFSA‐DDI_noRA	0.9599	0.9870	0.9606	0.9459	0.9757	0.9829
RFSA‐DDI_M_noRA	0.9610	0.9862	0.9613	0.9466	**0.9775**	0.9821
**RFSA‐DDI**	**0.9624**	**0.9891**	**0.9628**	**0.9519**	0.9740	**0.9856**

*Note*: The optimal result in each metric is indicated in bold.

Abbreviations: ACC, accuracy; AP, average precision; AUC, area under the curve; F1, F1‐score; Prec, precision; Rec, recall; RFSA‐DDI, substructure‐aware graph neural network incorporating relation features for drug–drug interaction prediction; RFSA‐DDI_M, RFSA‐DDI replaces graph self‐adaptive pooling with global mean pooling; RFSA‐DDI_M_noRA, RFSA‐DDI replaces graph self‐adaptive pooling with global mean pooling and removes the drug information and relation information interactive perception process in the RSAM module; RFSA‐DDI_noRA, RFSA‐DDI removes the drug information and relation information interactive perception process in the RSAM module.

**TABLE 6:** Ablation comparison results of RFSA‐DDI and its deformation on the DrugBank dataset under the inductive setting for $M_{s1}$ Partition (new drug—new drug).

Method	ACC	AUC	F1	Prec	Rec	AP
RFSA‐DDI_M	0.6385	0.7160	0.5155	0.7813	0.3846	0.7324
RFSA‐DDI_noRA	0.6298	0.7107	0.4769	**0.8126**	0.3375	0.7279
RFSA‐DDI_M_noRA	0.6310	0.7037	0.5051	0.7665	0.3766	0.7145
**RFSA‐DDI**	**0.6673**	**0.7600**	**0.5691**	0.8075	**0.4394**	**0.7703**

*Note*: The optimal result in each metric is indicated in bold.

Abbreviations: ACC, accuracy; AP, average precision; AUC, area under the curve; F1, F1‐score; Prec, precision; Rec, recall; RFSA‐DDI, substructure‐aware graph neural network incorporating relation features for drug–drug interaction prediction; RFSA‐DDI_M, RFSA‐DDI replaces graph self‐adaptive pooling with global mean pooling; RFSA‐DDI_M_noRA, RFSA‐DDI replaces graph self‐adaptive pooling with global mean pooling and removes the drug information and relation information interactive perception process in the RSAM module; RFSA‐DDI_noRA, RFSA‐DDI removes the drug information and relation information interactive perception process in the RSAM module.

**TABLE 7:** Ablation comparison results of RFSA‐DDI and its deformation on the DrugBank dataset under the inductive setting for $M_{s2}$ Partition (new drug—old drug).

Method	ACC	AUC	F1	Prec	Rec	AP
RFSA‐DDI_M	0.7346	0.8164	0.6938	0.8200	0.6013	0.8238
RFSA‐DDI_noRA	0.7339	0.8059	0.6943	0.8158	0.6043	0.8156
RFSA‐DDI_M_noRA	0.7264	0.8034	0.6801	**0.8186**	0.5817	0.8136
RFSA‐DDI	**0.7559**	**0.8327**	**0.7305**	0.8152	**0.6618**	**0.8339**

*Note*: The optimal result in each metric is indicated in bold.

Abbreviations: ACC, accuracy; AP, average precision; AUC, area under the curve; F1, F1‐score; Prec, precision; Rec, recall; RFSA‐DDI, substructure‐aware graph neural network incorporating relation features for drug–drug interaction prediction; RFSA‐DDI_M, RFSA‐DDI replaces graph self‐adaptive pooling with global mean pooling; RFSA‐DDI_M_noRA, RFSA‐DDI replaces graph self‐adaptive pooling with global mean pooling and removes the drug information and relation information interactive perception process in the RSAM module; RFSA‐DDI_noRA, RFSA‐DDI removes the drug information and relation information interactive perception process in the RSAM module.

### 2.7 Visual inspection

To address the credibility issue of applying the model to DDI prediction tasks, we present visual examples of DDI predictions on the DrugBank dataset that provide insights into the potential causes of DDIs. Specifically, we select two atoms in the drug pair (*d*_*x*_,*d*_*y*_) that have the maximum interaction odds $\mu_{p}^{\left(x\right)}$ and $\mu_{q}^{\left(y\right)}$ with the other drug in the drug pair, as derived from Equation (16). These atoms can be considered as the cores of the most significant substructures of drugs. Their size and form will be decided by the maximum importance score of substructures represented by Equation (8) (e.g., if the attention score is highest in the third iteration, the substructure with a radius of three is established).

**FIGURE 5:**
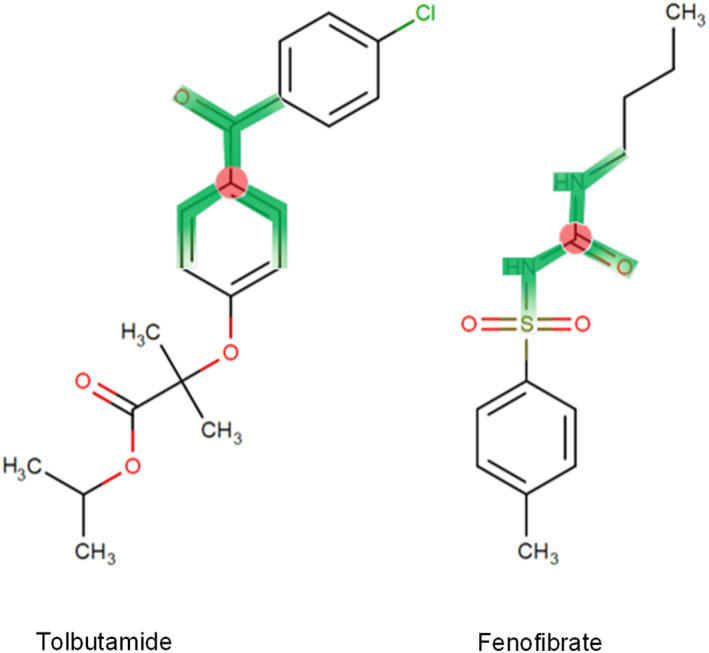
The visual representation of the critical substructures for DDI prediction between Fenofibrate and Tolbutamide. The core of the most essential substructure and its receptive field are depicted in red and green hues correspondingly. DDI, drug–drug interaction.

**FIGURE 6:**
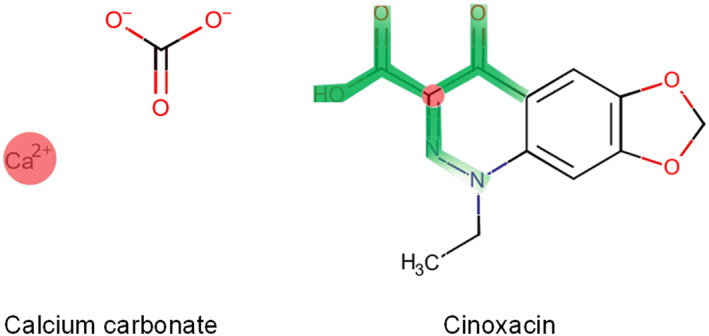
Visual inspection of DDI prediction between calcium carbonate and cinoxacin. DDI, drug–drug interaction.

We analyze the interaction between fenofibrate (a fibrate drug) and tolbutamide (a sulfonylurea drug). The central atomic importance of Fenofibrate and Tolbutamide is 0.5574 and 0.6808, respectively. When the iteration number *t* = 2, the radius attention scores of the two molecules reach their maximum values, which are 0.1431 and 0.1371, respectively. As illustrated in Figure 5, we can clearly detect that the RFSA‐DDI identifies the sulfonamide group of tolbutamide as having a significant impact on the interaction between the drugs, consistent with the fact that the concurrent use of sulfonylureas and fibrate increase the incidence of hypoglycemia [45]. Fibrate drugs can increase the blood concentration of sulfonylurea drugs and thereby elevate the risk of hypoglycemia through mechanisms such as the inhibition of CYP2C9 enzyme metabolism. Figure 6 is the example of the interaction between cinoxacin (a fluoroquinolone) and calcium carbonate (an antacid). Calcium carbonate has the highest radius attention score at *t* = 1, with a central atomic importance of 0.6099. On the other hand, cinoxacin achieves the highest radius attention score at *t* = 2, with a central atomic importance of 0.5063. Calcium carbonate significantly reduces the absorption of fluoroquinolone drugs in the human body [46]. This is consistent with the fact that the Ca ions in calcium carbonate can form complexes with fluoroquinolone antibiotics containing the carbonyl oxygen and the carboxylic acid, leading to absorption barriers for cinoxacin and thus potentially contributing to treatment failure in anti‐infective therapy.

### 2.8 Case study

To further verify the prediction effectiveness of our model, we train the model using the training set from the DrugBank dataset containing 86 interaction types. We then estimate drug pairs that are not within the training set using the RFSA‐DDI model and sort the prediction scores. We select the top 10 drug pairs related to types 1*, 2*, and 3* with the greatest prediction scores for validation using the Interactions Checker tool provided by the website of Drugbank. The specific interactions involved in these three types are shown in Table 8, and the verification results are shown in Table 9.

**TABLE 8:** The specific interactions involved in the three interaction types.

Type number	Specific description
1*	Drug 1 may increase the bronchoconstrictive activities of drug 2
2*	Drug 1 may increase the myopathic rhabdomyolysis activities of drug 2
3*	Drug 1 may decrease the excretion rate of drug 2, resulting in a higher serum level

**TABLE 9:** Results of predictions for selected top 10 drug pairs.

Drug 1	Drug 2	Interaction	Results
Ethchlorvynol	Triprolidine	2*	√
Deflazacort	Cyclosporine	1*	√
Etomidate	Difenoxin	2*	√
Mycophenolate mofetil	Oxyphenbutazone	2*	√
Avanafil	Lopinavir	3*	√
Fentanyl	Methyclothiazide	2*	√
Pitavastatin	Boceprevir	3*	√
Rufinamide	Prazepam	2*	√
Benzydamine	Medrysone	2*	√
Silodosin	Desipramine	3*	√

As evident from the results, all the top 10 drug pairs based on prediction scores exhibit documented interactions, thereby demonstrating the accuracy and robustness of our model’s predictions. For instance, the interaction 1* between Deflazacort and Cyclosporine was correctly predicted, indicating that when used together, the former may increase the bronchoconstrictory activities of the latter. The interaction between Ethchlorvynol and Triprolidine was also correctly predicted, suggesting that when used together, the former may increase the myopathic rhabdomyolysis activities of the latter (2*). Meanwhile, the prediction for the interaction 3* between Silodosin and Desipramine, which indicated that Silodosin may decrease the excretion rate of Desipramine, resulting in a higher serum level, was also successful. As a whole, these results emphasized the positive role of RFSA‐DDI in drug repurposing and prospective interaction testing before clinical co‐medication.

## 3 DISCUSSION

The effective prediction of DDIs can provide more accurate and reliable guidance for drug development and clinical practice. In this work, we offer a unique substructure‐aware DL model called RFSA‐DDI, which integrates relational features to predict potential DDIs between drugs. Compared to existing methods, test results demonstrate that our model displays greater performance in transductive along with inductive settings. The model directly leverages the structural information of drugs for feature extraction and inputs the extracted node and bond embedding information into the GSP‐DMPNN and RSAM modules to extract crucial substructure information and generate drug and relation feature representations. GSP‐DMPNN utilizes GSAPooling pooling operation to comprehensively consider the relation between node feature information and local information of the graph, which can more accurately calculate the importance score of substructures with different radii, thereby improving the quality of extracted substructure feature representations and guiding subsequent drug feature extraction work. The RSAM module utilizes the extracted substructure feature representations to obtain the initial drug graph feature representations. Based on this, relation embedding is incorporated for interactive perception and updating of drug and interaction feature representations. Furthermore, critical substructures involved in drug interactions are identified and given higher weights, allowing each drug to communicate which substructures are truly relevant to another drug, better understanding the reasons behind the interactions. In addition, our visual inspection also demonstrated that the model can effectively detect critical substructures involved in DDIs, capturing valuable structural information about drugs more accurately. Our approach integrates the characterization of drug graphs with the relation characterization of interaction types, optimizing the feature extraction process. This enables a more comprehensive understanding of drug molecules’ characteristics and generates more valuable feature representations, consequently enhancing the reliability and interpretability of the model.

Our method leverages the information of drug interactions solely through individual DDIs tuples without considering the potential positive impact of incorporating relation information from all DDIs tuples holistically. Inspired by Yu et al. [11], our future work will further generate an interaction network based upon the drug and interaction information in the dataset, where nodes indicate drugs and edges indicate interaction relations. By exploiting the network topology information, our method will provide additional clues for DDI prediction on a global scale, intending to boost the predictive performance of the model.

## 4 MATERIALS AND METHODS

In this section, we offer a full introduction to our proposed RFSA‐DDI. Firstly, we define the problem to be addressed and present the necessary symbol notations. Next, we describe the input representation and elaborate on the relevant implementation details and all computational stages involved in our approach. The complete framework is represented in Figure 1.

### 4.1 Dataset

In our experiments, we employed two authentic datasets, the DrugBank and the Twosides dataset, to assess the performance of RFSA‐DDI.

1. DrugBank: DrugBank [47] is a bioinformatics database that provides extensive drug information. It comprises 1706 drugs along with 191,808 DDI triplets. There are 86 types of interactions that clarify how one drug impacts the metabolism of another drug. For example, when Amoxapine is used in combination with Abiraterone, the metabolism level of Amoxapine decreases. In this dataset, each drug pair has one and only one interaction.
2. Twosides: Twosides is a dataset derived from the original Twosides dataset through screening and preprocessing by Zitnik et al. [48]. It consists of 645 drugs, with 4,576,287 DDI triplets and 963 types of interactions. These interactions are all related to adverse effects, such as diarrhea, skin allergies. In this dataset, each drug pair may involve multiple drug interactions.

### 4.2 Methods

#### 4.2.1 Problem formulation

The DDI prediction assignment involves constructing a model that takes a drug pair containing two different drugs, along with a specific interaction as input, and outputs the potential probability of the specified drug interaction *r* occurring for this drug pair. It can be stated as a function *f*: D×D×R→[0,1]. That is, given a DDIs dataset $M={\left\{{\left(d_{x},d_{y},r\right)}_{j}\right\}}_{j=1}^{J}$, where *d*_*x*_ and *d*_*y*_ represent drugs within drug datasets D and *r* is from DDI relation datasets R, the goal is to find an approximate computational method for function *f*.

#### 4.2.2 Input representation

The model will be fed with a DDI triplet (*d*_*x*_,*d*_*y*_,*r*). Both drugs, represented by Simplified Molecular Input Line Entry Specification strings, are converted into mol objects using the RDKit. The mol object is a depiction of the drug molecule in the form of a graph *G* = (*V*,*E*), where $V={\left\{v_{i}\right\}}_{i=1}^{n}$ signifies the set of nodes in the drug molecule graph and $E={\left\{{\left(e_{ij}=\left(v_{i},v_{j}\right)\right)}_{s}\right\}}_{s=1}^{m}$ signifies the set of edges. Each node *v*_*i*_ in the molecular graph has an initial feature vector $x_{v_{i}}\in R^{d}$, and each edge *e*_*ij*_ also includes an initial vector $x_{e_{ij}}\in R^{d^{\prime }}$. The node feature vector includes seven properties such as atom type, degree, hybridization. The edge feature vector includes three properties such as bond type, conjugation status. At the original phase, the graph is regarded as undirected. Therefore, the edges *e*_*ij*_ and *e*_*ji*_ have the same feature representation, that is, $x_{e_{ij}}=x_{e_{ji}}$.

#### 4.2.3 Message passing neural network

**FIGURE 7:**
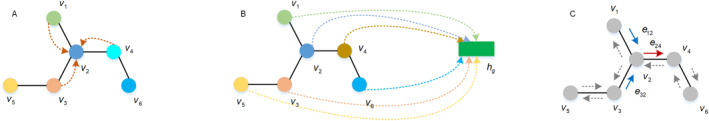
Description of graph embedding. (A) Message passing stage in MPNN. (B) Readout stage in MPNN. (C) Diagram of edge‐level message passing.

Provided a graph *G* = (*V*,*E*), if we want to convert its feature information into a feature vector representation, we can adopt the classic GNN—MPNN for information aggregation, updating, and extraction based on the graph structure information. The MPNN consists of two main stages: message passing and readout. The message passing stage is employed to update node states by aggregating information from surrounding nodes within graph *G*. The readout stage basically delivers a graph‐level feature vector by applying a readout function to aggregate node‐level features from across the entire graph *G*, as illustrated in Figure 7A,B.

Specifically, the message passing comprises *T* steps. At step *t*, with the aim of taking the updated feature $x_{v_{p}}^{\left(t\right)}\in R^{d}$ of node *v*_*p*_, we utilize the message function *M* ^(*t*)^(·) and the node update function *U* ^(*t*)^(·) to aggregate features from adjacent nodes:

(1) $$m_{v_{p}}^{\left(t\right)}=\sum_{v_{q}\in N\left(v_{p}\right)}M^{\left(t\right)}\left(x_{v_{p}}^{\left(t-1\right)},x_{v_{q}}^{\left(t-1\right)},x_{e_{pq}}\right),$$

(2) $$x_{v_{p}}^{\left(t\right)}=U^{\left(t\right)}\left(x_{v_{p}}^{\left(t-1\right)},m_{v_{p}}^{\left(t\right)}\right),$$

where N(p) signifies the set of adjacent nodes of node *v*_*p*_ in the graph *G*, $m_{v_{p}}^{\left(t\right)}$ represents the message received by node *v*_*p*_ at *t* step, $x_{e_{pq}}\in R^{d^{\prime }}$ represents the edge feature.

After completing the last step *T* of the message passing stage, we aggregate the updated node features obtained after step *T* to extract the graph‐level feature representation *h*_*g*_ of graph *G*:

(3) $$h_{g}=Z\left(x_{v_{p}}^{T}:v_{p}\in V\right),$$

where *Z*(·) is the readout function, *V* signifies the set of nodes in the graph *G*.

However, the aforementioned GNN suffers from limitations when applied to drug feature extraction and substructure generation. Firstly, the GNN extracts fixed‐size substructures with a predetermined number of layers. It is insufficient to capture the global structure of the molecules [37]. Secondly, there is redundancy in accessing nodes during the process of node information aggregation, which affects the quality of feature generation. Moreover, the drug properties are determined and ultimately influenced by the critical functional groups (substructures) of the drugs, making it necessary to effectively differentiate the importance of substructures during the readout stage. Therefore, some studies [34, 35, 37] have attempted to update the key‐level features for message passing, enabling messages to flow in only one direction, effectively reducing redundancy problems. Attention mechanisms have also been designed to evaluate the importance of substructures, optimizing the process of substructure extraction. However, there is still potential for refinement in current approaches to better utilize graph structure information and enhance the predictive performance of DDIs.

#### 4.2.4 Directed message passing neural network with substructure attention mechanism based on graph self‐adaptive pooling

The GSP‐DMPNN is designed for more accurate and flexible substructure extraction. In the iteration *t*, the GSP‐DMPNN extracts substructures with a radius of *t*, and obtains a self‐adaptive sized molecular substructure feature centered around an atom by calculating the weighted sum of different radius substructures, as shown in Figure 2.

Similar to the way D‐MPNN [49] updates node features through message passing, GSP‐DMPNN also utilizes edge‐based message passing instead of node‐based, as shown in Figure 7C. Similar to existing GNNs, each node *v*_*p*_ has a node‐level hidden feature $h_{v_{p}}^{\left(t\right)}$ during each layer of message passing, where $h_{v_{p}}^{\left(0\right)}=x_{v_{p}}$. Each edge *e*_*pq*_ also has an edge‐level hidden feature $h_{e_{pq}}^{\left(t\right)}$. The initialization of the edge‐level features is as follows:

(4) $$h_{e_{pq}}^{\left(0\right)}=\frac{1}{3}\left(W_{p}x_{v_{p}}+W_{q}x_{v_{q}}+W_{pq}x_{e_{pq}}\right),$$

where *W*_*p*_,*W*_*q*_ ∈ *R* ^*f*×*d*^ and $W_{pq}\in R^{f\times d^{\prime }}$ are learnable weight matrices.

At step *t*, we first calculate the edge‐level feature $h_{e_{pq}}^{\left(t\right)}$. We then leverage the notion of substructure attention to adaptively allocate objective attention weights for each substructure. A novel structure‐aware global pooling method is employed to obtain a graph representation at the bond level, that is, the line graph feature representation $g_{b}^{\left(t\right)}\in R^{f}$ is as follows:

(5) $$g_{b}^{\left(t\right)}=\sum_{p=1}^{n}\sum_{v_{q}\in N\left(v_{p}\right)}\beta_{qp}h_{e_{qp}}^{\left(t\right)},$$

where we employ the more efficient GSAPooling to compute the importance vector *β*_*qp*_. Different from SAGPooling, which solely considers the local information of the graph and calculates node importance scores based on the processed data from the GraphConv function, GSAPooling incorporates linearly transformed data of the node features. This integration of node feature information and graph local information enables the calculation of node importance scores, resulting in better representations of the overall graph features. The computation of the importance vector *β*_*qp*_ in GSAPooling is as follows:

(6) $$\beta_{qp}=\text{soft}\max\left(\alpha \text{GCNConv}\left(A,X\right)+\left(1-\alpha \right)FC\left(X\right)\right),$$

where *X* denotes the feature matrix at the bond level, and *A* represents the corresponding bond‐level adjacency matrix. The element value in the adjacency matrix is altered to one if two bonds share a common vertex. softmax(·) is applied to normalize the importance scores. GCNConv(·) denotes the aggregation operation on the neighbor features of nodes within the line graph, extracting local structural information and obtaining a 1‐dimensional vector representation. *FC*(·) refers to a fully connected layer that performs a linear transformation on the node features, extracting node‐specific information and also obtaining a 1‐dimensional vector representation. The weight *α* is a user‐defined hyperparameter, which we set to 0.6 in this work.

Next, we compute attention scores for each layer based on the line graph feature representation $g_{b}^{\left(t\right)}$ gained from step *t*:

(7) $$e^{\left(t\right)}=c^{\left(t\right)}\cdot \tanh\left(W_{b}g_{b}^{\left(t\right)}+b_{e}\right),$$

where $c^{\left(t\right)}\in R^{f^{\prime }}$ represents a learnable transformation vector at step *t*. The weight matrix $W_{b}\in R^{f^{\prime }\times f}$ and bias term *b*_*e*_ are used to preprocess the graph‐level representation $g_{b}^{\left(t\right)}$, and tanh(·) is an activation function. Dot product operation is denoted by (·). To allow for a more objective comparison of the coefficients across different layers, we apply the softmax function to normalize the generated scores *e* ^(*t*)^ for each step *t*:

(8) $$\lambda^{\left(t\right)}=\frac{\exp\left(e^{\left(t\right)}\right)}{\sum_{k=1}^{T}\;\exp\left(e^{\left(t\right)}\right)},$$

where *λ* ^(*t*)^ ∈ *R* ^1^ signifies the significance assigned to substructures within a radius of *t*. After the last iteration step *T*, the final representation of edge *e*_*pq*_ can be derived, which flexibly aggregates information from the hidden features of each layer using *λ*. The equation is given as follows:

(9) $$h_{e_{pq}}=\sum_{t=1}^{T}\lambda^{\left(t\right)}h_{e_{pq}}^{\left(t\right)}.$$

Finally, we aggregate all the incoming bond‐level features to node *v*_*p*_, resulting in the final node‐level feature $s_{v_{p}}$, which represents the local structural information centered around node *v*_*p*_:

(10) $$s_{v_{p}}=f_{s}\left(x_{v_{p}}+\sum_{v_{q}\in N\left(v_{p}\right)}h_{e_{qp}}\right),$$

where the function *f*_*s*_(·) is a non‐linear function executed with a multi‐layer perceptron (MLP), which farther deals with the aggregated feature of nodes.

Through the aforementioned steps of the GSP‐DMPNN, substructure representations centered around each node are obtained. In the process of generating substructure representations, the aggregation for neighbor node features has been further optimized, taking into account the local information, topological characteristics of the graph, and features of nodes and bonds in a more reasonable manner.

#### 4.2.5 Substructure‐aware interaction module incorporating relation features

In the current research on incorporating substructure information into DDIs prediction, the majority of studies extract drug substructures or graph‐level representations and then randomly embed vectors for relations, which are directly input into the final DDIs prediction task. However, they overlook the significant impact of relation embedding on identifying latent drug characteristics. And they fail to consider optimizing drug feature representations by incorporating specific drug interaction relations during the construction of drug representations. Moreover, directly inputting randomly embedded relation vectors into the prediction module can potentially affect the final DDI identification performance of the model.

Based on this idea, we schedule to construct RSAM. Building upon the initial drug graph feature representations, we incorporate relation embedding for refinement. Simultaneously, the relation embedding also integrates drug information for optimization, enabling mutual perception and updates between both entities. Consequently, the model implements the DRIP and leverages richer information and more discriminative features to enhance DDI prediction performance.

**FIGURE 8:**
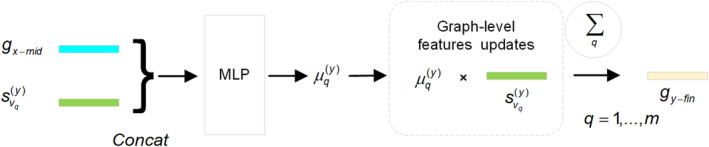
Overall computational steps of graph‐level feature update process based on substructure‐drug perception (SDUP). Concat, concatenation operation concatenation operation; MLP, multilayer perceptron; SDUP, substructure‐drug perception.

Furthermore, in order to construct high‐quality drug characteristic representations for DDIs prediction tasks, based on DRIP, we should align the module architecture with the chemical reaction process of drug pairs. That is, identify crucial functional substructures of drugs and assign them higher weights. We hypothesize that the structural information of one drug in a drug pair can be used to identify crucial substructures of the other drug. Hence, RSAM will implement the SDUP. SDUP determines the importance coefficients of substructures by evaluating the interaction between drug substructures and the other drug contained within the drug pair and aggregates the weighted substructure representations to generate the final drug representation, as illustrated in Figure 8.

Specifically, for a provided drug pair (*d*_*x*_,*d*_*y*_), we initially learn the global feature of the drug molecule. In this regard, we employ a computation method that differs from the commonly used readout functions in GNNs to obtain graph‐level feature representations. We opt for a more reasonable strategy by selecting a node importance coefficient allocation approach. For a drug *d*_*x*_, we extract the feature representation for the node within the drug molecular structure graph *G*_*x*_ based on the GSP‐DMPNN. These node representations provide information about the substructures centered around each node. Next, we aggregate all the node features in the graph by applying a learnable coefficient *β*_*p*_ to weight each feature, which can be interpreted as its importance. As a result, we extract the initial representation of *d*_*x*_:

(11) $$g_{x\_\text{init}}=\sum_{p=1}^{n}\beta_{p}s_{v_{p}}^{\left(x\right)}.$$

The coefficient *β*_*p*_ can also be computed with the involvement of GSAPooling. The initial graph‐level representation $g_{y\_\text{init}}$ can also be described using a similar Equation (11). The given drug pair (*d*_*x*_,*d*_*y*_) involves the relation type *r*_*z*_ ∈ *R* ^*f*^ which is randomly initialized at this stage. After obtaining the initial graph feature representation for the drug pair, we integrate the drug information with the relation embedding to perform individual feature optimization, resulting in transitional feature representations for both drugs and relations:

(12) $$g_{x\_\text{mid}}=g_{x\_\text{init}}+f_{h}\left(o\right),$$

(13) $$g_{y\_\text{mid}}=g_{y\_\text{init}}+f_{t}\left(o\right),$$

(14) $$r_{z\_\text{new}}=r_{z}+f_{r}\left(o\right),$$

(15) $$o=\text{Concat}\left(W_{h}g_{x\_\text{init}},W_{t}g_{y\_\text{init}},r_{z}\right),$$

where *W*_*h*_,*W*_*t*_ ∈ *R* ^*f*×*f*^ denotes a learnable weight matrix utilized for linear transformation towards the drug’s initial feature representation involved in the concatenation operation. *f*_*h*_(·), *f*_*t*_(·) and *f*_*r*_(·) denotes a nonlinear transformation implemented using MLP. Then, we assess the interaction odds between the *q*th substructure in drug *d*_*y*_ and drug *d*_*x*_:

(16) $$\mu_{q}^{\left(y\right)}=\text{soft}\max\left(f_{\mu }\left(\text{Concat}\left(W_{u}g_{x\_\text{mid}},W_{v}s_{v_{q}}^{\left(y\right)}\right)\right)\right),$$

where the function *f*_*μ*_(·) is a nonlinear function used to process the concatenated data of each substructure feature representation in *d*_*y*_ and the global representation in *d*_*x*_. The weight matrix $W_{u},W_{v}\in R^{f^{\prime }\times f}$ is learnable, and $\mu_{q}^{\left(y\right)}$ can be interpreted as the significance of the substructure centered around the *q*th atom in *d*_*y*_. At last, the final global representation of *d*_*y*_ can be produced through weighted aggregation:

(17) $$g_{y\_fin}=\sum_{q=1}^{m}\mu_{q}^{\left(y\right)}s_{v_{q}}^{\left(y\right)}.$$

RSAM leverages the structural information within *d*_*x*_ to highlight the relevant weights of crucial substructures in *d*_*y*_ that impact the interaction, reducing the weights of unnecessary substructures and emphasizing significant features to optimize the global representation of *d*_*y*_. The final graph‐level representation $g_{x\_fin}$ of *d*_*x*_ can be established via similar calculation steps described in Equations ((11), (12), (13), (14), (15), (16), (17))–(17).

#### 4.2.6 DDI prediction

Given a DDI tuple (*d*_*x*_,*d*_*y*_,*r*), the DDI prediction can be represented as the joint probability of the tuple:

(18) $$P\left(d_{x},d_{y},r\right)=\sigma \left(r_{z\_\text{new}}\cdot f_{w}\left(\text{Concat}\left(g_{x\_f\text{in}},g_{y\_f\text{in}}\right)\right)\right),$$

where $r_{z\_\text{new}}\in R^{f}$, obtained from Equation (14), is a learnable vector of the relation type *r* and *f*_*w*_(·) denotes a nonlinear function. *σ* denotes the sigmoid function used to constrain the drug interaction probability within the range [0,1]. The model optimization will be performed by minimizing the cross‐entropy loss function [25] provided as:

(19) $$Loss=-\frac{1}{J}\sum_{j=1}^{J}y_{j}\;\log\left(p_{j}\right)+\left(1-y_{j}\right)\log\left(1-p_{j}\right),$$

where *y*_*j*_ = 1 signifies the existence of interaction about the drug pair (*d*_*x*_,*d*_*y*_). *p*_*j*_ signifies the predicted odds of interaction about the DDI tuple, which is calculated by applying Equation (18).

## AUTHOR CONTRIBUTIONS

**Liangcheng Dong**: Conceptualization; formal analysis; investigation; methodology; project administration; resources; validation; visualization; writing – original draft. **Baoming Feng**: Software. **Zengqian Deng**: Investigation. **Jinlong Wang**: Conceptualization. **Peihao Ni**: Data curation; visualization. **Yuanyuan Zhang**: Conceptualization; formal analysis; methodology; supervision.

## CONFLICT OF INTEREST STATEMENT

The authors Liangcheng Dong, Baoming Feng, Zengqian Deng, Jinlong Wang, Peihao Ni, and Yuanyuan Zhang declare that they have no conflict of interest or financial conflicts to disclose.

## ETHICS STATEMENT

All procedures performed in studies involving animals were in accordance with the ethical standards of the institution or practice at which the studies were conducted, and with the 1964 Helsinki declaration and its later amendments or comparable ethical standards.

## Data Availability

All instructions and codes for our experiments are available at the website of Github (donglc506/RFSA‐DDI).
